# Costs and Healthcare Utilization of Heart Disease by COVID-19 Diagnosis and Race and Ethnicity

**DOI:** 10.1016/j.focus.2024.100285

**Published:** 2024-10-06

**Authors:** Jun Soo Lee, Yidan (Xue) Zhang, Lisa M. Pollack, Feijun Luo

**Affiliations:** 1 Division for Heart Disease and Heart Disease Prevention, Centers for Disease Control and Prevention, Atlanta, Georgia; 2 ASRT, Inc., Atlanta, Georgia

**Keywords:** Heart disease, medical costs, healthcare utilization, racial and ethnic disparities, COVID-19, Medicaid

## Abstract

**Introduction:**

Heart disease poses a significant health and economic burden in the U.S., with considerable variations in outcomes across different racial and ethnic groups. The COVID-19 pandemic has further highlighted the disparities in healthcare utilization and costs associated with heart disease.

**Methods:**

The authors used the 2021 Merative MarketScan Medicaid claims database to estimate the medical costs and healthcare utilization associated with heart disease by racial and ethnic groups and COVID-19 diagnosis status. This study focused on individuals aged ≥18 years continuously enrolled in a noncapitated insurance plan in 2021. The outcome measures included total medical expenditures and healthcare utilization, including the numbers of emergency department visits and inpatient admissions and length of inpatient stay. The authors employed a generalized linear model with a family of gamma and log links for medical costs, and a negative binomial regression was used for healthcare utilization. Three-way interactions of heart disease, COVID-19 diagnosis, and race and ethnicity categories were implemented after adjusting for age, sex, and comorbidities. The authors reported average marginal effects with 95% CIs.

**Results:**

Among 1,008,166 Medicaid beneficiaries, 8% had heart disease in 2021. The cost associated with heart disease was $10,819 per beneficiary in 2021 (95% CI=10,292; 11,347; *p*<0.001). The cost was $15,840 (95% CI=14,389; 17,291; *p*<0.001) for non-Hispanic Black individuals; $9,945 (95% CI=9,172; 10,718; *p*<0.001) for non-Hispanic White; and $8,511 (95% CI=7,490; 9,531; *p*<0.001) for Hispanic individuals. Individuals with a COVID-19 diagnosis ($19,638) had $9,541 (95% CI=7,049; 12,032; *p*<0.001) higher costs associated with heart disease than those without COVID-19 ($10,098) (*p*<0.001). Individuals with heart disease had higher numbers of emergency department visits (0.937 per beneficiary, 95% CI=0.913; 0.960), inpatient admissions (0.463 per beneficiary, 95% CI=0.455; 0.471), and average length of stay (2.541 days per admission, 95% CI=2.405; 2.677) than those without heart disease.

**Conclusions:**

The study's findings showed that costs and healthcare utilization associated with heart disease are substantial in all racial and ethnic groups and the highest among non-Hispanic Black individuals. Furthermore, individuals with a COVID-19 diagnosis had approximately 2 times higher costs associated with heart disease than individuals without a COVID-19 diagnosis.

## HIGHLIGHTS

• Significant racial disparities in heart disease (HD) costs among Medicaid beneficiaries.
• COVID-19 diagnosis doubles HD costs, highlighting pandemic's impact on health care.
• Patients with HD exhibit higher emergency department visits, inpatient admissions, and longer stays.
• Non-Hispanic Black individuals face highest HD costs, underscoring disparities.
• Findings inform policy for reducing HD burden and addressing COVID-19 impact.

## INTRODUCTION

Heart disease (HD) is the leading cause of mortality in the U.S., responsible for 1 in every 5 deaths in 2021.^1^^,^^2^ Although HD is preventable and treatable, approximately 29 million adults had the condition in 2018.^1^ The average annual medical cost of HD was $117 billion nationwide in 2018–2019, making it the fifth most expensive among 22 major chronic diseases.^1^

Individuals with HD tend to have worse health outcomes after a coronavirus disease 2019 (COVID-19) diagnosis than those without HD. Underlying HD is associated with worse respiratory symptoms associated with COVID-19 that may require admission into intensive care units and/or utilization of invasive ventilation devices.^3^ In addition, oxygen deprivation during a COVID-19 diagnosis may cause heart damage, increasing the risk of acute cardiac events such as heart failure and myocardial infarction.^4^, ^5^, ^6^ In addition, individuals with HD may be more likely to delay or avoid health care for fear of contracting severe acute respiratory syndrome coronavirus 2 (SARS-CoV-2), the virus that causes COVID-19, leading to poorer postinfection outcomes.^7^

Both HD and COVID-19 affect different racial and ethnic groups unevenly. For instance, HD mortality is higher among non-Hispanic Black than among non-Hispanic White individuals.^8^ During the COVID-19 pandemic, non-Hispanic Black and Hispanic individuals experienced greater challenges in accessing health care than did their non-Hispanic White counterparts.^7^ Although overall mortality from HD increased in the first year of the pandemic, the increase was greater among non-Hispanic Black, non-Hispanic Asian, and Hispanic individuals than among non-Hispanic White individuals.^9^

To date, no research has examined the relationship between COVID-19 diagnosis and the cost of HD, and only a few studies documented variations in the cost of HD by race and ethnicity.^10^^,^^11^ One study found that Hispanic adults incurred greater costs from specific evidence-based structural HD interventions than non-Hispanic White adults, despite having poorer access to these interventions.^10^ Another study found that HD was associated with higher medical costs and greater inpatient treatment utilization for non-Hispanic Black adults than non-Hispanic White adults.^11^ However, neither study had a comparison group of individuals without HD to allow for the estimation of incremental cost and healthcare utilization attributable to HD. This study fills this gap by examining medical costs and utilization of various types of health care associated with HD by COVID-19 diagnosis, including by race and ethnicity. Because HD disproportionately affects socioeconomically disadvantaged populations,^12^, ^13^, ^14^ this study focused on individuals enrolled in Medicaid, a government-funded insurance program for individuals with limited financial resources.

## METHODS

### Study Sample

The authors used the Merative MarketScan Multi-State Medicaid Databases in 2021. The database came from 7 U.S. states and contains individuals with noncapitated (i.e., fee-for-service) and capitated (i.e., managed-care) insurance plans. Individual-level claim information includes out-of-pocket costs, insurance payments, records of emergency department (ED) visits, inpatient admissions, outpatient visits, pharmacy prescriptions, and service dates. Medicaid claims also include patients’ demographics, including age, sex, and race and ethnicity.^15^ Using the history of claims, researchers can identify patients’ comorbidities and COVID-19 diagnoses. This study was a secondary data analysis using deidentified information and considered nonresearch. Therefore, a review by the Centers for Disease Control and Prevention's IRB was unnecessary.

The authors included individuals aged ≥18 years, without dual eligibility for Medicare, continuously enrolled in Medicaid in 2021, and without a pregnancy-related diagnosis (Appendix Table 1, available online).^16^ The authors further restricted the sample to individuals with noncapitated health insurance in 2021 because managed-care (or capitated insurance) plans do not have accurate payment and healthcare utilization information. Individuals with HD include those whose claim records had at least 1 inpatient admission or 2 outpatient visits (at least 30 days apart) containing the HD diagnosis (Appendix Table 1, available online) in 2021.

### Measures

The main outcomes were (1) all-cause total medical costs (i.e., sum of individuals’ out-of-pocket costs and insurance payments) and (2) healthcare utilization (including the numbers of ED visits, inpatient admissions, outpatient visits, and pharmacy prescriptions). The authors also examined total length of stay from all inpatient admissions (days) and average length of stay per inpatient admission (days). All measures are presented per individual in 2021.

All models adjusted for individuals’ age (18–34 [reference], 35–44, 45–54, 55–64, 65–74, 75–84, and ≥85 years), race and ethnicity (non-Hispanic White [reference], non-Hispanic Black, Hispanic, and all other racial and ethnic groups [owing to confidentiality and data user agreements, the authors cannot report more detailed race and ethnicity information]), COVID-19 diagnosis status (identified using ICD-10-CM of U07.1), and Quan Charlson comorbidity conditions,^17^ excluding acute myocardial infarction and congestive heart failure conditions because they are directly related to HD. All comorbidity conditions were defined as indicators if individuals had at least 1 inpatient admission or 2 outpatient visits (30 days intervals) and contained the corresponding diagnosis in 2021.^17^

### Statistical Analysis

Differences in means of continuous variables were tested using the Wilcoxon nonparametric rank-sum test, and differences in proportions of categorical variables were tested using the Pearson's chi-square test for individuals with and without HD.

For total medical costs, the authors used a generalized linear model with a gamma distribution and a log link function. For healthcare utilization and length of stay outcome variables, the authors used a negative binomial model. For all models, the authors interacted the HD indicator with race and ethnicity and COVID-19 diagnosis status to test differences by COVID-19 diagnosis status for all race and ethnicity groups.

As a sensitivity analysis and to account for excessive zeros in costs, the authors used a 2-part model for total medical costs, where the first part is a logit model, and the second part is a generalized linear model with a gamma distribution and a log link function. In addition, to exclude potential extreme values that may affect the estimates, the authors excluded the top and bottom 1% of total medical costs.

All models adjusted for the control variables. The authors reported the predicted outcomes for individuals with and without HD and average marginal effects with a 95% CI. In addition, the authors presented the differences in predicted outcomes and average marginal effects with 95% CI by COVID-19 diagnosis status. SEs and 95% CIs were estimated using the delta method, as implemented in Stata 17 with the margins command. To determine statistical significance between race and ethnicity groups, the authors compared the 95% CIs; differences were considered statistically significant if the CIs did not overlap. This method was applied to assess the significance of the average marginal effects associated with HD.

The authors used *p*<0.05 to indicate statistical significance for 2-sided testing. The authors conducted all the analyses using Stata SE statistical software, Version 17.0 (StataCorp, College Station, TX), in March–July 2023.

## RESULTS

**Figure 1:**
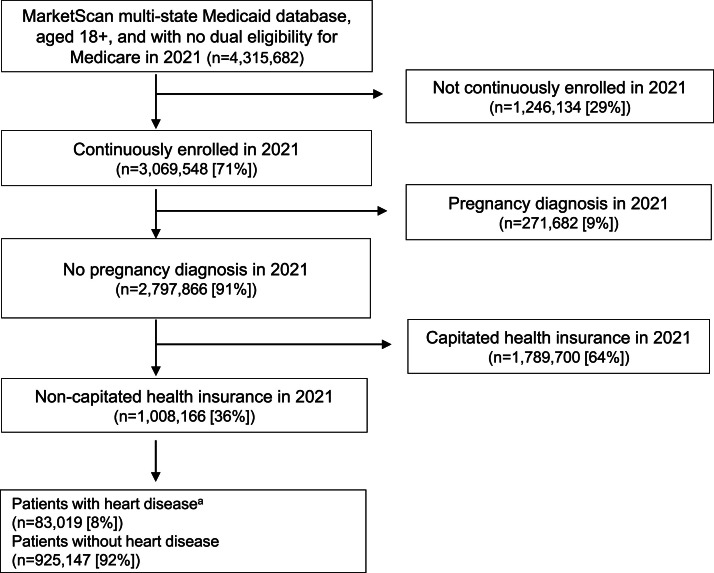
Study sample selection of patients diagnosed with heart disease, MarketScan Multi-State Medicaid Database, 2021.^a^ ^a^Patients with heart disease were identified if patients had at least 1 inpatient claim or 2 outpatient claims with 30-day intervals of heart disease (ICD-10-CM=I00-I09, I11, I13, and I20-I51).

Of the 1,008,166 individuals included in the sample (Figure 1), 83,019 (8%) had HD in 2021 (Table 1). Individuals with HD (mean [SD] age=54.5 [13.9] years) were older than those without HD (mean [SD] age=39.3 [14.9] years) (Table 1). Individuals with HD were less likely to be female (51.38% vs 58.6%; *p<*0.001) and more likely to be non-Hispanic White (47.54% vs 46.29%; *p<*0.001) or non-Hispanic Black (22.66% vs 18.42%; *p<*0.001) than those without HD. Individuals with HD had higher percentages of diagnoses with COVID-19 (16.21% vs 6.79%; *p<*0.001) and other comorbidities (all *p<*0.001) in 2021 than those without HD.

**Table 1:** Summary Statistics by Heart Disease Status, MarketScan Multi-State Medicaid Database, 2021^a^

	All	With heart disease	Without heart disease
Study sample	N=1,008,166 (100%)	*n*=83,019 (8.2%)	*n*=925,147 (91.8%)
Age, years, mean (SD)	40.5 (15.4)	54.5 (13.9)	39.3 (14.9)^⁎⁎⁎^
Age groups, years, *n* (%)			
18–34	419,180 (41.58%)	7,907 (9.52%)	411,273 (44.45%)^⁎⁎⁎^
35–44	200,854 (19.92%)	9,456 (11.39%)	191,398 (20.69%)^⁎⁎⁎^
45–54	160,432 (15.91%)	18,275 (22.01%)	142,157 (15.37%)^⁎⁎⁎^
55–64	177,293 (17.59%)	34,337 (41.36%)	142,956 (15.45%)^⁎⁎⁎^
65–74	30,864 (3.06%)	7,153 (8.62%)	23,711 (2.56%)^⁎⁎⁎^
75–84	13,587 (1.35%)	3,936 (4.74%)	9,651 (1.04%)^⁎⁎⁎^
≥85	5,956 (0.59%)	1,955 (2.35%)	4,001 (0.43%)^⁎⁎⁎^
Female, *n* (%)	584,770 (58.00%)	42,656 (51.38%)	542,114 (58.60%)^⁎⁎⁎^
Race categories, *n* (%)			
Non-Hispanic White	467,744 (46.40%)	39,468 (47.54%)	428,276 (46.29%)^⁎⁎⁎^
Non-Hispanic Black	189,214 (18.77%)	18,810 (22.66%)	170,404 (18.42%)^⁎⁎⁎^
Hispanic	152,571 (15.13%)	7,047 (8.49%)	145,524 (15.73%)^⁎⁎⁎^
Other race groups	198,637 (19.70%)	17,694 (21.31%)	180,943 (19.56%)^⁎⁎⁎^
COVID-19 diagnosis in 2021	76,257 (7.56%)	13,458 (16.21%)	62,799 (6.79%)^⁎⁎⁎^
Comorbidities, *n* (%)			
Cerebrovascular	11,755 (1.17%)	5,938 (7.15%)	5,817 (0.63%)^⁎⁎⁎^
Peripheral vascular	22,171 (2.20%)	12,777 (15.39%)	9,394 (1.02%)^⁎⁎⁎^
Dementia	8,217 (0.82%)	3,028 (3.65%)	5,189 (0.56%)^⁎⁎⁎^
Chronic pulmonary disease	92,510 (9.18%)	29,620 (35.68%)	62,890 (6.80%)^⁎⁎⁎^
Rheumatic disease	10,417 (1.03%)	2,627 (3.16%)	7,790 (0.84%)^⁎⁎⁎^
Peptic ulcer disease	3,019 (0.30%)	1,421 (1.71%)	1,598 (0.17%)^⁎⁎⁎^
Mild liver disease	26,101 (2.59%)	8,852 (10.66%)	17,249 (1.86%)^⁎⁎⁎^
Diabetes without chronic complication	99,770 (9.90%)	29,210 (35.18%)	70,560 (7.63%)^⁎⁎⁎^
Diabetes with chronic complication	40,562 (4.02%)	17,169 (20.68%)	23,393 (2.53%)^⁎⁎⁎^
Hemiplegia or paraplegia	12,450 (1.23%)	3,590 (4.32%)	8,860 (0.96%)^⁎⁎⁎^
Renal disease	25,262 (2.51%)	13,584 (16.36%)	11,678 (1.26%)^⁎⁎⁎^
Any malignancy	20,188 (2.00%)	5,658 (6.82%)	14,530 (1.57%)^⁎⁎⁎^
Moderate or severe liver disease	4,037 (0.40%)	1,769 (2.13%)	2,268 (0.25%)^⁎⁎⁎^
Metastatic solid tumor	4,690 (0.47%)	1,664 (2.00%)	3,026 (0.33%)^⁎⁎⁎^
AIDS/HIV	6,743 (0.67%)	1,292 (1.56%)	5,451 (0.59%)^⁎⁎⁎^

*Note:* ***p<0.001.

a The authors used the Wilcoxon nonparametric rank-sum test to test the differences in means for continuous variables and the Pearson's chi-square test to test differences in proportions for categorical variables by heart disease status.

Table 2 presents per-person total medical costs associated with HD, overall and stratified by COVID-19 diagnosis and race and ethnicity. Overall, individuals with HD ($23,790; 95% CI=23,247; 24,334) had $10,819 (95% CI=10,292; 11,347; *p<*0.001) higher total medical costs than those without HD ($12,971; 95% CI=12,778; 13,164).

**Table 2:** Total Medical Costs Associated With Heart Disease by COVID-19 Diagnosis and Race/Ethnicity, 2021^a^

Race/ethnicity	All	Without COVID-19 diagnosis	With COVID-19 diagnosis	With COVID-19 versus without COVID-19b
All races				
Without heart disease	12,971	12,012	24,688	12,675***
	(12,778; 13,164)	(11,852; 12,173)	(23,829; 25,546)	(11,896; 13,454)
With heart disease	23,790	22,110	44,326	22,216***
	(23,247; 24,334)	(21,574; 22,646)	(41,864; 46,788)	(19,738; 24,694)
Difference^c^	10,819***	10,098***	19,638***	9,541***
	(10,292; 11,347)	(9,566; 10,629)	(17,195; 22,081)	(7,049; 12,032)
Observation	1,008,166	931,909	76,257	1,008,166
Non-Hispanic White				
Without heart disease	14,451	13,443	27,234	13,792***
	(14,227; 14,675)	(13,249; 13,636)	(26,087; 28,381)	(12,700; 14,883)
With heart disease	24,396	22,827	44,280	21,454***
	(23,625; 25,167)	(22,052; 23,602)	(40,726; 47,835)	(17,838; 25,069)
Difference^c^	9,945***	9,384***	17,046***	7,662***
	(9,172; 10,718)	(8,603; 10,166)	(13,414; 20,679)	(3,952; 11,372)
Observation	467,744	433,531	34,213	467,744
Non-Hispanic Black				
Without heart disease	15,885	14,315	34,572	20,257***
	(15,497; 16,273)	(13,996; 14,634)	(32,331; 36,813)	(18,101; 22,413)
With heart disease	31,725	28,965	64,579	35,614***
	(30,253; 33,198)	(27,505; 30,425)	(57,650; 71,508)	(28,597; 42,630)
Difference^c^	15,840***	14,650***	30,007***	15,357***
	(14,389; 17,291)	(13,199; 16,101)	(22,931; 37,082)	(8,146; 22,568)
Observation	189,214	174,549	14,665	189,214
Hispanic				
Without heart disease	5,314	5,007	8,635	3,628***
	(5,217; 5,410)	(4,916; 5,097)	(8,129; 9,140)	(3,123; 4,134)
With heart disease	13,824	13,081	21,868	8,787***
	(12,808; 14,841)	(12,024; 14,137)	(18,180; 25,556)	(4,952; 12,622)
Difference^c^	8,511***	8,074***	13,233***	5,159**
	(7,490; 9,531)	(7,014; 9,134)	(9,517; 16,950)	(1,296; 9,023)
Observation	152,571	139,661	12,910	152,571

*Note:* ***p<0.001, **p<0.01, and *p<0.05.

a A generalized linear model with a family of gamma and log links with was used. All models were adjusted to patients’ age, sex, race categories (for all only), COVID-19 infection status, and comorbidities. The average predicted total medical costs in U.S. dollars with 95% CI for individuals with and without heart disease are reported. The robust SEs were used. The differences and 95% CI in the predicted total medical costs for individuals with heart disease and without heart disease were reported. Interaction terms of heart disease indicator, race categories, and COVID-19 diagnosis indicator were used to calculate the predicted values for the non-Hispanic White, non-Hispanic Black, and Hispanic individuals by COVID-19 diagnosis status. Differences in the average marginal effects associated with heart disease between race and ethnicity groups were deemed statistically significant if their 95% CIs did not overlap. ^b^Differences with 95% CI in the predicted values and average marginal effects of heart disease by COVID-19 diagnosis status are reported.

c Difference in costs between individuals with heart disease and those without heart disease.

Among individuals without a COVID-19 diagnosis, those with HD ($22,110 [95% CI=21,574; 22,646]) had $10,098 (95% CI=9,566; 10,629; *p<*0.001) higher total medical costs than those without HD ($12,012 [95% CI=11,852; 12,173]). Among individuals with a COVID-19 diagnosis, those with HD ($44,326 [95% CI=41,864; 46,788]) had $19,638 (95% CI=17,195; 22,081; *p<*0.001) higher total medical costs than those without HD ($24,688 [95% CI=23,829; 25,546]). Therefore, a COVID-19 diagnosis increased medical costs associated with HD by $9,541 (95% CI=7,049; 12,032; *p<*0.001) (Table 2).

By race and ethnicity, the overall excess costs associated with HD were $9,945 (95% CI=9,172; 10,718; *p<*0.001) for non-Hispanic White individuals; $15,840 (95% CI=14,389; 17,291; *p<*0.001) for non-Hispanic Black individuals; and $8,511 (95% CI=7,490; 9,531; *p<*0.001) for Hispanic individuals.

By COVID-19 diagnosis and race and ethnicity, non-Hispanic White individuals with a COVID-19 diagnosis ($17,046 [95% CI=13,414; 20,679]) had $7,662 (95% CI=3,952; 11,372; *p<*0.001) higher medical costs associated with HD than those without a COVID-19 diagnosis ($9,384 [95% CI=8,603; 10,166]). Non-Hispanic Black individuals with a COVID-19 diagnosis ($30,007 [95% CI=22,931; 37,082]) had $15,357 (95% CI=8,146; 22,568; *p<*0.001) higher medical costs associated with HD than those without a COVID-19 diagnosis ($14,650 [95% CI=13,199; 16,101]). Hispanic individuals with COVID-19 diagnosis ($13,233 [95% CI=9,517; 16,950]) had $5,159 (95% CI=1,296; 9,023; *p<*0.01) higher total medical costs associated with HD than those without COVID-19 diagnosis ($8,074 [95% CI=7,014; 9,134]).

Tables 3 and 4 present per-person number of ED visits and inpatient admissions associated with HD by COVID-19 diagnosis and race and ethnicity. Overall, the number of per-person ED visits associated with HD was 0.937 (95% CI=0.913, 0.960; *p<*0.001) (Table 3). The number of per-person ED visits associated with HD did not significantly differ by COVID-19 diagnosis for all races and ethnicities combined; however, it significantly differed by COVID-19 diagnosis for individual racial and ethnic groups. Non-Hispanic White individuals with a COVID-19 diagnosis (1.018 [95% CI=0.888, 1.148]) had 0.143 (95% CI=0.010, 0.275; *p<*0.05) more ED visits associated with HD than those without a COVID-19 diagnosis (0.875 [95% CI=0.845, 0.906]). On the other hand, non-Hispanic Black (−0.309, 95% CI= −0.520, −0.097; *p<*0.01) and Hispanic individuals with a COVID-19 diagnosis (−0.303, 95% CI= −0.547, −0.0603; *p<*0.05) had fewer ED visits associated with HD than those without a COVID-19 diagnosis.

**Table 3:** Average Number of Emergency Department Visits and Inpatient Admissions Associated With Heart Disease by COVID-19 Diagnosis and Race/Ethnicity, 2021^a^: Number of Emergency Department Visits

Race/ethnicity	All	Without COVID-19 diagnosis	With COVID-19 diagnosis	With COVID-19 versus without COVID-19^b^
All races				
Without heart disease	0.867	0.763	2.128	1.365***
	(0.863, 0.871)	(0.760, 0.767)	(2.098, 2.158)	(1.335, 1.395)
With heart disease	1.803	1.702	3.042	1.340***
	(1.781, 1.826)	(1.679, 1.725)	(2.957, 3.126)	(1.253, 1.427)
Difference^c^	0.937***	0.939***	0.914***	−0.0248
	(0.913, 0.960)	(0.915, 0.962)	(0.824, 1.003)	(−0.116, 0.0666)
Observation	1,008,166	931,909	76,257	1,008,166
Non-Hispanic White				
Without heart disease	0.834	0.742	2.002	1.260***
	(0.828, 0.839)	(0.737, 0.746)	(1.961, 2.042)	(1.219, 1.301)
With heart disease	1.720	1.617	3.020	1.403***
	(1.690, 1.749)	(1.587, 1.647)	(2.896, 3.143)	(1.276, 1.529)
Difference^c^	0.886***	0.875***	1.018***	0.143*
	(0.856, 0.916)	(0.845, 0.906)	(0.888, 1.148)	(0.0100, 0.275)
Observation	467,744	433,531	34,213	467,744
Non-Hispanic Black				
Without heart disease	1.009	0.866	2.709	1.843***
	(0.999, 1.019)	(0.858, 0.874)	(2.625, 2.794)	(1.759, 1.928)
With heart disease	2.075	1.956	3.491	1.535***
	(2.025, 2.125)	(1.905, 2.008)	(3.303, 3.679)	(1.340, 1.729)
Difference^c^	1.066***	1.090***	0.782***	−0.309**
	(1.015, 1.118)	(1.038, 1.143)	(0.576, 0.988)	(−0.520, −0.0970)
Observation	189,214	174,549	14,665	189,214
Hispanic				
Without heart disease	0.843	0.745	1.894	1.148***
	(0.834, 0.851)	(0.738, 0.753)	(1.835, 1.952)	(1.089, 1.207)
With heart disease	1.765	1.694	2.539	0.845***
	(1.694, 1.836)	(1.619, 1.768)	(2.314, 2.763)	(0.609, 1.081)
Difference^c^	0.923***	0.949***	0.645***	−0.303*
	(0.851, 0.994)	(0.873, 1.024)	(0.413, 0.877)	(−0.547, −0.0603)
Observation	152,571	139,661	12,910	152,571

*Note:* ***p<0.001, **p<0.01, and *p<0.05.

a A negative binomial model was used. All models were adjusted to patients’ age, sex, race categories (for all only), COVID-19 infection status, and comorbidities. The average predicted total medical costs in U.S. dollars with 95% CI for individuals with and without heart disease are reported. The robust SEs were used. The differences and 95% CIs in the predicted total medical costs for individuals with heart disease and without heart disease were reported. Interaction terms of heart disease indicator, race categories, and COVID-19 diagnosis indicator were used to calculate the predicted values for the non-Hispanic White, non-Hispanic Black, and Hispanic individuals by COVID-19 diagnosis status. Differences in the average marginal effects associated with heart disease between race and ethnicity groups were deemed statistically significant if their 95% CIs did not overlap.

b Differences with 95% CI in the predicted values and average marginal effects of heart disease by COVID-19 diagnosis status are reported.

c Difference in costs between individuals with heart disease and those without heart disease.

**Table 4:** Average Number of Emergency Department Visits and Inpatient Admissions Associated With Heart Disease by COVID-19 Diagnosis and Race/Ethnicity, 2021^a^: Number of Inpatient Admissions

Race/ethnicity	All	Without COVID-19 diagnosis	With COVID-19 diagnosis	With COVID-19 versus without COVID-19^b^
All races				
Without heart disease	0.119	0.0967	0.386	0.289***
	(0.117, 0.120)	(0.0957, 0.0978)	(0.375, 0.396)	(0.279, 0.299)
With heart disease	0.582	0.515	1.397	0.882***
	(0.574, 0.590)	(0.507, 0.523)	(1.354, 1.440)	(0.839, 0.924)
Difference^c^	0.463***	0.418***	1.011***	0.593***
	(0.455, 0.471)	(0.411, 0.426)	(0.969, 1.053)	(0.551, 0.635)
Observation	1,008,166	931,909	76,257	1,008,166
Non-Hispanic White				
Without heart disease	0.124	0.104	0.377	0.273***
	(0.123, 0.126)	(0.103, 0.106)	(0.364, 0.390)	(0.260, 0.286)
With heart disease	0.569	0.511	1.296	0.785***
	(0.558, 0.580)	(0.501, 0.522)	(1.239, 1.353)	(0.727, 0.842)
Difference^c^	0.444***	0.407***	0.919***	0.512***
	(0.434, 0.455)	(0.396, 0.418)	(0.861, 0.976)	(0.454, 0.570)
Observation	467,744	433,531	34,213	467,744
Non-Hispanic Black				
Without heart disease	0.161	0.125	0.581	0.455***
	(0.157, 0.164)	(0.123, 0.128)	(0.551, 0.610)	(0.427, 0.484)
With heart disease	0.766	0.664	1.977	1.313***
	(0.746, 0.786)	(0.645, 0.684)	(1.866, 2.088)	(1.201, 1.424)
Difference^c^	0.606***	0.539***	1.396***	0.857***
	(0.586, 0.626)	(0.520, 0.558)	(1.285, 1.508)	(0.744, 0.970)
Observation	189,214	174,549	14,665	189,214
Hispanic				
Without heart disease	0.0605	0.0493	0.181	0.132***
	(0.0589, 0.0620)	(0.0480, 0.0507)	(0.170, 0.192)	(0.121, 0.143)
With heart disease	0.422	0.381	0.869	0.488***
	(0.403, 0.441)	(0.362, 0.400)	(0.786, 0.952)	(0.403, 0.573)
Difference^c^	0.362***	0.331***	0.688***	0.356***
	(0.343, 0.381)	(0.312, 0.351)	(0.604, 0.771)	(0.271, 0.442)
Observation	152,571	139,661	12,910	152,571

*Note:* ***p<0.001, **p<0.01, and *p<0.05.

a A negative binomial model was used. All models were adjusted to patients’ age, sex, race categories (for all only), COVID-19 infection status, and comorbidities. The average predicted total medical costs in U.S. dollars with 95% CIs for individuals with and without heart disease are reported. The robust SEs were used. The differences and 95% CIs in the predicted total medical costs for individuals with heart disease and without heart disease were reported. Interaction terms of heart disease indicator, race categories, and COVID-19 diagnosis indicator were used to calculate the predicted values for the non-Hispanic White, non-Hispanic Black, and Hispanic individuals by COVID-19 diagnosis status. Differences in the average marginal effects associated with heart disease between race and ethnicity groups were deemed statistically significant if their 95% CIs did not overlap.

b Differences with 95% CIs in the predicted values and average marginal effects of heart disease by COVID-19 diagnosis status are reported.

c Difference in costs between individuals with heart disease and those without heart disease.

Overall, individuals with HD (0.582 [95% CI=0.574, 0.590]) had 0.463 (95% CI=0.455, 0.471; *p<*0.001) more inpatient admissions than those without HD (0.119 [95% CI=0.117, 0.120]) (Table 4). Individuals with a COVID-19 diagnosis (1.011 [95% CI=0.969, 1.053]) had 0.593 (95% CI=0.551, 0.635; *p<*0.001) more inpatient admissions associated with HD than those without a COVID-19 diagnosis (0.418 [95% CI=0.411, 0.426]). Non-Hispanic White (0.512, 95% CI=0.454, 0.570; *p<*0.001), non-Hispanic Black (0.857, 95% CI=0.744, 0.970; *p<*0.001), and Hispanic individuals with a COVID-19 diagnosis (0.356, 95% CI=0.271, 0.442; *p<*0.001) had more inpatient admissions associated with HD than those without COVID-19 diagnosis.

Tables 5 and 6 show the per-person total inpatient days and average length of inpatient admissions associated with HD. Overall, individuals with HD (8.379 [95% CI=7.751, 9.008]) had about a 7-day longer total inpatient stay (6.892 days, 95% CI=6.359, 7.426; *p<*0.001) and about a 3-day longer average inpatient stay (2.541 days, 95% CI=2.405, 2.677; *p<*0.001) per admission than individuals without HD (1.487 [95% CI=1.373, 1.601]). Individuals with a COVID-19 diagnosis had longer total inpatient stays associated with HD (22.12 days, 95% CI=18.75, 25.49; *p<*0.001) and longer average inpatient stays per admission associated with HD (6.506 days, 95% CI=5.605, 7.406; *p<*0.001) than individuals without a COVID-19 diagnosis.

**Table 5:** Total and Average Length of Stay for Inpatient Admissions With Heart Disease by COVID-19 Diagnosis and Race/Ethnicity, 2021^a^: Total Length of Stay for Inpatient Admissions

Race/ethnicity	All	Without COVID-19 diagnosis	With COVID-19 diagnosis	With COVID-19 versus without COVID-19^b^
All races				
Without heart disease	1.487	1.017	7.230	6.213***
	(1.373, 1.601)	(0.949, 1.085)	(6.502, 7.958)	(5.544, 6.882)
With heart disease	8.379	6.236	34.57	28.33***
	(7.751, 9.008)	(5.809, 6.663)	(30.58, 38.56)	(24.60, 32.07)
Difference^c^	6.892***	5.219***	27.34***	22.12***
	(6.359, 7.426)	(4.847, 5.591)	(23.79, 30.89)	(18.75, 25.49)
Observation	1,008,166	931,909	76,257	1,008,166
Non-Hispanic White				
Without heart disease	1.375	0.999	6.148	5.150***
	(1.281, 1.469)	(0.940, 1.057)	(5.523, 6.774)	(4.568, 5.731)
With heart disease	7.484	5.787	28.99	23.20***
	(6.911, 8.057)	(5.373, 6.201)	(24.89, 33.09)	(19.25, 27.15)
Difference^c^	6.109***	4.788***	22.84***	18.05***
	(5.601, 6.616)	(4.412, 5.164)	(19.01, 26.67)	(14.31, 21.79)
Observation	467,744	433,531	34,213	467,744
Non-Hispanic Black				
Without heart disease	3.023	2.025	14.89	12.87***
	(2.711, 3.334)	(1.830, 2.220)	(12.89, 16.90)	(11.00, 14.74)
With heart disease	14.80	11.22	57.44	46.22***
	(13.12, 16.48)	(9.940, 12.50)	(47.00, 67.88)	(36.23, 56.22)
Difference^c^	11.78***	9.193***	42.55***	33.36***
	(10.32, 13.24)	(8.053, 10.33)	(32.77, 52.33)	(23.83, 42.88)
Observation	189,214	174,549	14,665	189,214
Hispanic				
Without heart disease	0.382	0.254	1.766	1.512***
	(0.359, 0.406)	(0.243, 0.266)	(1.572, 1.960)	(1.323, 1.701)
With heart disease	4.449	3.255	17.36	14.11***
	(3.910, 4.988)	(2.854, 3.656)	(12.95, 21.78)	(9.704, 18.52)
Difference^c^	4.066***	3.000***	15.60***	12.60***
	(3.533, 4.600)	(2.601, 3.399)	(11.21, 19.98)	(8.213, 16.98)
Observation	152,571	139,661	12,910	152,571

*Note:* ***p<0.001, **p<0.01, and *p<0.05.

a A negative binomial model was used. All models were adjusted to patients’ age, sex, race categories (for all only), COVID-19 infection status, and comorbidities. The average predicted total medical costs in U.S. dollars with 95% CIs for individuals with and without heart disease are reported. The robust SEs were used. The differences and 95% CIs in the predicted total medical costs for individuals with heart disease and without heart disease were reported. Interaction terms of heart disease indicator, race categories, and COVID-19 diagnosis indicator were used to calculate the predicted values for the non-Hispanic White, non-Hispanic Black, and Hispanic individuals by COVID-19 diagnosis status. Differences in the average marginal effects associated with heart disease between race and ethnicity groups were deemed statistically significant if their 95% CIs did not overlap.

b Differences with 95% CIs in the predicted values and average marginal effects of heart disease by COVID-19 diagnosis status are reported.

c Difference in costs between individuals with heart disease and those without heart disease.

**Table 6:** Total and Average Length of Stay for Inpatient Admissions With Heart Disease by COVID-19 Diagnosis and Race/Ethnicity, 2021^a^: Average Length of Stay per Inpatient Admission per Patient

Race/ethnicity	All	Without COVID-19 diagnosis	With COVID-19 diagnosis	With COVID-19 versus without COVID-19^b^
All races				
Without heart disease	0.665	0.492	2.779	2.288***
	(0.632, 0.697)	(0.472, 0.512)	(2.574, 2.985)	(2.099, 2.477)
With heart disease	3.206	2.541	11.33	8.793***
	(3.046, 3.366)	(2.425, 2.657)	(10.30, 12.36)	(7.813, 9.774)
Difference^c^	2.541***	2.049***	8.555***	6.506***
	(2.405, 2.677)	(1.946, 2.152)	(7.625, 9.485)	(5.605, 7.406)
Observation	1,008,166	931,909	76,257	1,008,166
Non-Hispanic White				
Without heart disease	0.645	0.496	2.525	2.029***
	(0.616, 0.673)	(0.478, 0.514)	(2.330, 2.720)	(1.845, 2.212)
With heart disease	3.013	2.469	9.911	7.442***
	(2.848, 3.178)	(2.338, 2.599)	(8.751, 11.07)	(6.309, 8.575)
Difference^c^	2.368***	1.972***	7.386***	5.413***
	(2.218, 2.518)	(1.850, 2.095)	(6.283, 8.489)	(4.324, 6.503)
Observation	467,744	433,531	34,213	467,744
Non-Hispanic Black				
Without heart disease	1.180	0.865	4.926	4.061***
	(1.096, 1.263)	(0.811, 0.919)	(4.390, 5.461)	(3.558, 4.563)
With heart disease	4.689	3.761	15.73	11.97***
	(4.297, 5.081)	(3.444, 4.079)	(13.31, 18.16)	(9.622, 14.32)
Difference^c^	3.510***	2.896***	10.81***	7.913***
	(3.165, 3.854)	(2.608, 3.184)	(8.503, 13.12)	(5.638, 10.19)
Observation	189,214	174,549	14,665	189,214
Hispanic				
Without heart disease	0.234	0.160	1.029	0.869***
	(0.223, 0.244)	(0.154, 0.165)	(0.936, 1.123)	(0.777, 0.962)
With heart disease	2.227	1.700	7.927	6.226***
	(1.999, 2.455)	(1.517, 1.883)	(6.163, 9.690)	(4.459, 7.994)
Difference^c^	1.993***	1.540***	6.897***	5.357***
	(1.767, 2.220)	(1.357, 1.723)	(5.140, 8.655)	(3.594, 7.120)
Observation	152,571	139,661	12,910	152,571

*Note:* ***p<0.001, **p<0.01, and *p<0.05.

a A negative binomial model was used. All models were adjusted to patients’ age, sex, race categories (for all only), COVID-19 infection status, and comorbidities. The average predicted total medical costs in U.S. dollars with 95% CIs for individuals with and without heart disease are reported. The robust SEs were used. The differences and 95% CIs in the predicted total medical costs for individuals with heart disease and without heart disease were reported. Interaction terms of heart disease indicator, race categories, and COVID-19 diagnosis indicator were used to calculate the predicted values for the non-Hispanic White, non-Hispanic Black, and Hispanic individuals by COVID-19 diagnosis status. Differences in the average marginal effects associated with heart disease between race and ethnicity groups were deemed statistically significant if their 95% CIs did not overlap.

b Differences with 95% CIs in the predicted values and average marginal effects of heart disease by COVID-19 diagnosis status are reported.

c Difference in costs between individuals with heart disease and those without heart disease.

Non-Hispanic White, non-Hispanic Black, and Hispanic individuals with a COVID-19 diagnosis had statistically significantly longer total inpatient stays and longer average inpatient stays per admission than those without COVID-19 (Tables 5 and 6). A COVID-19 diagnosis increased total length of stay for inpatient admission associated with HD by 18.05 days (95% CI=14.31, 21.79) for non-Hispanic White individuals, 33.36 days (95% CI=23.83, 42.88) for non-Hispanic Black individuals, and 12.60 days (95% CI=8.213, 16.98) for Hispanic individuals. A COVID-19 diagnosis increased average length of stay per inpatient admission associated with HD by 5.41 days (95% CI=4.32, 6.50) for non-Hispanic White individuals, 7.91 days (95% CI=5.64, 10.19) for non-Hispanic Black individuals, and 5.36 days (95% CI=3.59, 7.12) for Hispanic individuals.

Individuals with HD had a higher number of outpatient visits and pharmacy prescriptions than those without HD (Appendix Table 2, available online). The differences by COVID-19 diagnosis were statistically significant for all races and non-Hispanic White individuals for pharmacy prescriptions. However, the differences were not significant for the number of outpatient visits.

Appendix Tables 3 and 4 (available online) provide the total medical costs associated with HD using the 2-part model and keeping 1%–99% of the costs, respectively. The results using the 2-part model were similar to the main findings, indicating that the percentage of excess zeros for total medical costs was not large enough to affect the estimates. The costs associated with HD were slightly lower when keeping 1%–99% of the total medical costs.

## DISCUSSION

Using an administrative multistate Medicaid claims database from 2021, the authors estimated the medical costs and healthcare utilization associated with HD by COVID-19 diagnosis and race and ethnicity. Individuals with HD had higher total medical costs and healthcare utilization than those without HD. Individuals with a COVID-19 diagnosis had almost 2 times the total medical costs, double the number of inpatient admissions, and 4–5 times longer inpatient stays associated with HD than individuals without a COVID-19 diagnosis. By race and ethnicity, non-Hispanic Black individuals had higher total medical costs and healthcare utilization than non-Hispanic White individuals.

The results of this study showed that the all-cause excess medical cost associated with HD was $10,819 per adult. The authors could not find comparable estimates in prior studies; studies either did not have a comparison group and therefore did not look at the incremental medical costs associated with HD^11^^,^^18^ or focused on more acute HD, such as heart failure. For example, a 2020 systematic review found that the annual mean heart failure treatment costs were $29,118 (range: $14,226–$45,784) in 2019 U.S. dollars.^19^ Their numbers are higher than those in this study likely because their review focused on treatment costs for heart failure, which is an acute form of HD. In addition, the results of this study suggest that HD is associated with increased ED visits, inpatient admissions, inpatient length of stay, outpatient visits, and pharmacy prescriptions, which is consistent with findings in existing research.^19^, ^20^, ^21^, ^22^

This study is the first to estimate the medical costs and healthcare utilization for HD associated with COVID-19. The authors found that among individuals with HD, those with a COVID-19 diagnosis had substantially higher all-cause medical costs than individuals without a COVID-19 diagnosis. The differences in total medical costs were mainly driven by the higher number of inpatient admissions and the greater length of inpatient stay associated with HD among individuals with a COVID-19 diagnosis. Similarly, the authors observed that HD was associated with a greater increase in all-cause medical cost associated with COVID-19, driven by higher utilization of inpatient treatments among individuals with HD. A possible explanation of these differences in total medical costs and inpatient-related outcomes is that individuals with HD have a high likelihood of experiencing severe respiratory symptoms and acute cardiac events after a COVID-19 diagnosis, and these conditions typically require intensive treatment and around-the-clock monitoring.^3^^,^^6^^,^^23^^,^^24^ In addition, preexisting HD increases individuals’ risk of long COVID symptoms, potentially explaining the higher all-cause medical cost associated with HD among those with a COVID-19 diagnosis.^25^ Strategies such as enhancing the awareness about the link between COVID-19 and HD and improving vaccination among individuals with HD may have the potential to curb healthcare spending related to HD and COVID-19.^26^^,^^27^

In addition, the results of this study indicated that the medical cost associated with HD is substantially higher among non-Hispanic Black individuals than among non-Hispanic White individuals. In line with existing research,^28^, ^29^, ^30^ the authors found that non-Hispanic Black adults had significantly higher inpatient healthcare utilization than non-Hispanic White adults, which may explain the cost differences between the 2 racial groups. Reducing disparities in access to and quality of preventative and primary care services may help close HD-related inpatient treatment gaps between non-Hispanic Black and White individuals.^31^ Using hospital cost data from 2015 to 2017, a study found that addressing heart failure treatment disparities between non-Hispanic Black and White patients in 6 Southern states could save $61 million in hospitalizations in a year.^30^

Notably, a COVID-19 diagnosis increased medical costs associated with HD twice as much for non-Hispanic Black adults as for non-Hispanic White adults. This may be explained by the finding that non-Hispanic Black adults with a COVID-19 diagnosis had a substantially greater increase in inpatient admissions and in total length of inpatient stay than non-Hispanic White adults. Research has documented that non-Hispanic Black individuals have less access to evidence-based quality HD treatment than non-Hispanic White individuals.^10^^,^^28^ Because poorly treated HD may increase the risk of severe COVID-19 symptoms,^32^ among adults with HD, non-Hispanic Black individuals may be more susceptible to severe COVID-19 symptoms requiring inpatient treatment than non-Hispanic White individuals.

### Limitations

This study has several limitations. First, the MarketScan data include Medicaid enrollees in fee-for-service plans in only 5–13 states in the U.S. Therefore, these results may not be generalizable to the entire Medicaid population or non-Medicaid patients. Second, this study's reliance on the MarketScan database limits the ability to control for unmeasured confounding factors such as income, educational attainment, and state policies, which are not available in this data set. Although the authors controlled for observed confounders, unmeasured socioeconomic factors may still influence the observed associations. Third, the authors only included individuals continuously enrolled in Medicaid in 2021. However, a federal policy restricted states’ authority to disenroll Medicaid beneficiaries during the COVID-19 public health emergency (i.e., the maintenance of enrollment policy), and 71% of all enrollees in 2021 MarketScan data had continuous coverage.^33^^,^^34^ Fourth, following the MarketScan data user agreement, the authors were not able to study costs and healthcare utilization for non-Hispanic racial groups other than White and Black individuals. Fifth, given the cross-sectional study design, the authors could not consider the long-term costs associated with HD (e.g., those stemming from long COVID symptoms). Future studies may address this issue using longitudinal data.

## CONCLUSIONS

Using Medicaid claims database, the authors documented medical costs and healthcare utilization associated with HD by COVID-19 diagnosis and race and ethnicity in 2021. The costs associated with HD were significantly higher for individuals with a COVID-19 diagnosis than for those without the diagnosis. The authors found substantially higher costs and healthcare utilization associated with HD among non-Hispanic Black individuals than among non-Hispanic White individuals. These findings contribute to a better understanding of the cost of HD among adults with low income and how HD and COVID-19 together affect these costs. The cost estimates from this study can be used to measure the potential economic benefits (healthcare costs averted) of interventions effective in preventing COVID-19 among patients with HD, including those targeting various racial and ethnic groups. This information is crucial for economic evaluation, which compares the economic costs and benefits of effective interventions and informs the planning and prioritization of healthcare resource allocation.

## Appendix Supplementary materials

Image, application 1

## Footnotes

Supplementary material associated with this article can be found in the online version at doi:10.1016/j.focus.2024.100285.
