# Supplementary material for: Costs and Healthcare Utilization of Heart Disease by COVID-19 Diagnosis and Race and Ethnicity
Source: AJPM Focus. 2024 Oct 6;4(1):100285. doi: 10.1016/j.focus.2024.100285 (PMC11613426; doi:10.1016/j.focus.2024.100285)
Supplement: Supplementary file 1 [file mmc1.docx]

**Appendix Materials**

**Appendix Table 1.** ICD-10-CM diagnosis, procedure, and DRG codes for pregnancy and ICD-10-CM diagnosis codes for heart disease.

|  | ICD-10-CM | DRG | ICD-10-PCS |
| --- | --- | --- | --- |
| Pregnancy | O00-O99, O9A1-O9A5, Z33, Z34, Z36, Z37, Z3201, Z322, Z39, F53, A34 | 765, 766, 767, 768, 769, 770, 771, 772, 773, 775, 776, 777, 779, 780, 781, 782 | 10A0, 10D00Z0, 10D00Z1, 10D00Z2, 10D07Z3, 10D07Z4, 10D07Z5, 10D07Z6, 10D07Z7, 10D07Z8, 10E0XZZ |
| Heart Disease | I00-I09, I11, I13, I20-I51 | NA | NA |

Abbreviations: DRG, diagnosis-related group; ICD-10-CM, International Classification of Diseases, Tenth Revision, Clinical Modification; ICD-10-PCS, International Classification of Diseases, Tenth Revision, Procedure Coding System

NA indicates not available.

**Appendix Table 2. Number of outpatient visits and pharmacy prescriptions with heart disease by COVID-19 diagnosis and race ethnicity, 2021.^a^**

**Panel A. Number of outpatient visits**

|  | All | Without COVID-19 diagnosis | With COVID-19 diagnosis | With COVID-19 vs. Without COVID-19^c^ |
| --- | --- | --- | --- | --- |
| All races |  |  |  |  |
| Without heart disease | 33.53 | 32.71 | 43.61 | 10.90*** |
|  | (33.37 - 33.69) | (32.56 - 32.86) | (43.00 - 44.22) | (10.32 - 11.48) |
| With heart disease | 43.09 | 42.24 | 53.51 | 11.27*** |
|  | (42.63 - 43.56) | (41.75 - 42.73) | (52.14 - 54.88) | (9.824 - 12.71) |
| Difference^b^ | 9.559*** | 9.531*** | 9.899*** | 0.368 |
|  | (9.068 - 10.05) | (9.020 - 10.04) | (8.433 - 11.36) | (-1.163 - 1.898) |
| Observation | 1,008,166 | 931,909 | 76,257 | 1,008,166 |
| Non-Hispanic White |  |  |  |  |
| Without heart disease | 37.70 | 36.78 | 49.36 | 12.58*** |
|  | (37.49 - 37.91) | (36.57 - 36.98) | (48.44 - 50.28) | (11.68 - 13.49) |
| With heart disease | 43.66 | 42.77 | 54.98 | 12.22*** |
|  | (43.00 - 44.32) | (42.08 - 43.46) | (52.90 - 57.06) | (10.03 - 14.40) |
| Difference^b^ | 5.960*** | 5.987*** | 5.620*** | -0.367 |
|  | (5.265 - 6.655) | (5.263 - 6.711) | (3.370 - 7.870) | (-2.715 - 1.980) |
| Observation | 467,744 | 433,531 | 34,213 | 467,744 |
| Non-Hispanic Black |  |  |  |  |
| Without heart disease | 41.42 | 40.28 | 54.98 | 14.70*** |
|  | (41.07 - 41.78) | (39.94 - 40.63) | (53.39 - 56.57) | (13.12 - 16.27) |
| With heart disease | 58.24 | 56.89 | 74.33 | 17.43*** |
|  | (56.96 - 59.52) | (55.54 - 58.24) | (70.59 - 78.06) | (13.48 - 21.39) |
| Difference^b^ | 16.82*** | 16.61*** | 19.34*** | 2.737 |
|  | (15.50 - 18.14) | (15.22 - 17.99) | (15.32 - 23.37) | (-1.501 - 6.975) |
| Observation | 189,214 | 174,549 | 14,665 | 189,214 |
| Hispanic |  |  |  |  |
| Without heart disease | 15.78 | 15.38 | 20.05 | 4.674*** |
|  | (15.65 - 15.90) | (15.25 - 15.51) | (19.50 - 20.61) | (4.108 - 5.240) |
| With heart disease | 30.13 | 29.85 | 33.14 | 3.283* |
|  | (29.05 - 31.21) | (28.70 - 31.01) | (30.47 - 35.81) | (0.376 - 6.190) |
| Difference^b^ | 14.36*** | 14.47*** | 13.08*** | -1.391 |
|  | (13.26 - 15.45) | (13.31 - 15.64) | (10.35 - 15.81) | (-4.351 - 1.569) |
| Observation | 152,571 | 139,661 | 12,910 | 152,571 |

**Panel B. Number of any pharmacy prescriptions**

|  | All | Without COVID-19 diagnosis | With COVID-19 diagnosis | With COVID-19 vs. Without COVID-19^c^ |
| --- | --- | --- | --- | --- |
| All races |  |  |  |  |
| Without heart disease | 24.31 | 22.70 | 43.97 | 21.27*** |
|  | (24.15 - 24.46) | (22.56 - 22.84) | (43.24 - 44.70) | (20.58 - 21.96) |
| With heart disease | 37.16 | 35.78 | 54.00 | 18.21*** |
|  | (36.71 - 37.61) | (35.31 - 36.25) | (52.39 - 55.60) | (16.55 - 19.88) |
| Difference^b^ | 12.85*** | 13.08*** | 10.03*** | -3.055*** |
|  | (12.38 - 13.32) | (12.60 - 13.57) | (8.311 - 11.74) | (-4.825 - -1.284) |
| Observation | 1,008,166 | 931,909 | 76,257 | 1,008,166 |
| Non-Hispanic White |  |  |  |  |
| Without heart disease | 28.46 | 26.60 | 52.00 | 25.40*** |
|  | (28.24 - 28.67) | (26.41 - 26.79) | (50.86 - 53.13) | (24.30 - 26.50) |
| With heart disease | 42.13 | 40.67 | 60.68 | 20.01*** |
|  | (41.41 - 42.86) | (39.91 - 41.42) | (58.03 - 63.33) | (17.26 - 22.76) |
| Difference^b^ | 13.68*** | 14.07*** | 8.685*** | -5.386*** |
|  | (12.93 - 14.43) | (13.30 - 14.85) | (5.838 - 11.53) | (-8.326 - -2.446) |
| Observation | 467,744 | 433,531 | 34,213 | 467,744 |
| Non-Hispanic Black |  |  |  |  |
| Without heart disease | 26.92 | 24.72 | 53.04 | 28.32*** |
|  | (26.62 - 27.21) | (24.47 - 24.98) | (51.25 - 54.83) | (26.56 - 30.08) |
| With heart disease | 47.35 | 45.16 | 73.45 | 28.29*** |
|  | (46.16 - 48.54) | (43.92 - 46.39) | (69.17 - 77.72) | (23.86 - 32.72) |
| Difference^b^ | 20.43*** | 20.44*** | 20.40*** | -0.0316 |
|  | (19.22 - 21.64) | (19.18 - 21.69) | (15.83 - 24.98) | (-4.769 - 4.706) |
| Observation | 189,214 | 174,549 | 14,665 | 189,214 |
| Hispanic |  |  |  |  |
| Without heart disease | 10.49 | 9.827 | 17.60 | 7.772*** |
|  | (10.38 - 10.59) | (9.730 - 9.925) | (17.03 - 18.17) | (7.202 - 8.342) |
| With heart disease | 15.59 | 14.96 | 22.44 | 7.476*** |
|  | (14.95 - 16.23) | (14.29 - 15.63) | (20.34 - 24.54) | (5.274 - 9.678) |
| Difference^b^ | 5.109*** | 5.134*** | 4.838*** | -0.296 |
|  | (4.460 - 5.758) | (4.455 - 5.814) | (2.667 - 7.009) | (-2.569 - 1.977) |
| Observation | 152,571 | 139,661 | 12,910 | 152,571 |

^a^ A negative binomial model was used. All models were adjusted to patients’ age, sex, race categories (for all only), COVID-19 infection status, and comorbidities. The average predicted total medical costs with 95% CI for individuals with and without heart disease are reported. The robust standard errors were used. The differences and 95% CI in the predicted total medical costs for individuals with heart disease and without heart disease were reported. Interaction terms of heart disease indicator, race categories, and COVID-19 diagnosis indicator were used to calculate the predicted values for the NH-White, NH-Black, and Hispanic by COVID-19 diagnosis status.

^b^ Difference in costs for individuals with heart disease vs. those without heart disease. *** *P<*0.001, ** *P<*0.01, * *P<*0.05

^c^ Differences with 95% CI in the predicted values and average marginal effects of heart disease by COVID-19 diagnosis status are reported.

**Appendix Table 3. Total medical costs associated with heart disease by COVID-19 diagnosis and race ethnicity using Two-Part Model, 2021.^a^**

|  | All | Without COVID-19 diagnosis | With COVID-19 diagnosis | With COVID-19 vs. Without COVID-19^c^ |
| --- | --- | --- | --- | --- |
| All races |  |  |  |  |
| Without heart disease | 12,912 | 11,947 | 24,714 | 12,767*** |
|  | (12,725 - 13,100) | (11,791 - 12,102) | (23,852 - 25,575) | (11,979 - 13,555) |
| With heart disease | 23,563 | 21,926 | 43,568 | 21,642*** |
|  | (23,035 - 24,090) | (21,403 - 22,448) | (41,186 - 45,949) | (19,241 - 24,043) |
| Difference^b^ | 10,650*** | 9,979*** | 18,854*** | 8,875*** |
|  | (10,134 - 11,167) | (9,458 - 10,499) | (16,474 - 21,234) | (6,448 - 11,303) |
| Observation | 1,008,166 | 931,909 | 76,257 | 1,008,166 |
| Non-Hispanic White |  |  |  |  |
| Without heart disease | 14,364 | 13,361 | 27,069 | 13,707*** |
|  | (14,146 - 14,582) | (13,173 - 13,549) | (25,932 - 28,205) | (12,622 - 14,793) |
| With heart disease | 24,155 | 22,626 | 43,535 | 20,909*** |
|  | (23,403 - 24,908) | (21,868 - 23,383) | (40,084 - 46,986) | (17,396 - 24,422) |
| Difference^b^ | 9,791*** | 9,265*** | 16,466*** | 7,201*** |
|  | (9,034 - 10,549) | (8,499 - 10,030) | (12,927 - 20,006) | (3,585 - 10,818) |
| Observation | 467,744 | 433,531 | 34,213 | 467,744 |
| Non-Hispanic Black |  |  |  |  |
| Without heart disease | 16,056 | 14,392 | 35,857 | 21,465*** |
|  | (15,661 - 16,451) | (14,073 - 14,711) | (33,445 - 38,269) | (19,132 - 23,798) |
| With heart disease | 31,252 | 28,552 | 63,384 | 34,832*** |
|  | (29,813 - 32,691) | (27,123 - 29,981) | (56,615 - 70,153) | (27,973 - 41,692) |
| Difference^b^ | 15,196*** | 14,160*** | 27,527*** | 13,367*** |
|  | (13,771 - 16,621) | (12,736 - 15,584) | (20,544 - 34,510) | (6,251 - 20,484) |
| Observation | 189,214 | 174,549 | 14,665 | 189,214 |
| Hispanic |  |  |  |  |
| Without heart disease | 5,251 | 4,955 | 8,454 | 3,499*** |
|  | (5,158 - 5,344) | (4,867 - 5,042) | (7,970 - 8,937) | (3,015 - 3,983) |
| With heart disease | 13,826 | 13,098 | 21,700 | 8,602*** |
|  | (12,835 - 14,817) | (12,067 - 14,129) | (18,135 - 25,264) | (4,892 - 12,311) |
| Difference^b^ | 8,575*** | 8,143*** | 13,246*** | 5,103** |
|  | (7,580 - 9,570) | (7,109 - 9,178) | (9,654 - 16,839) | (1,365 - 8,840) |
| Observation | 152,571 | 139,661 | 12,910 | 152,571 |

^a^ A two-part model was used. The first part is a logit model and second part is a generalized linear model with a family of gamma and log links. All models were adjusted to patients’ age, sex, race categories (for all only), COVID-19 infection status, and comorbidities. The average predicted total medical costs with 95% CI for individuals with and without heart disease are reported. The robust standard errors were used. The differences and 95% CI in the predicted total medical costs for individuals with heart disease and without heart disease were reported. Interaction terms of heart disease indicator, race categories, and COVID-19 diagnosis indicator were used to calculate the predicted values for the NH-White, NH-Black, and Hispanic by COVID-19 diagnosis status.

^b^ Difference in costs for individuals with heart disease vs. those without heart disease. *** *P<*0.001, ** *P<*0.01, * *P<*0.05

^c^ Differences with 95% CI in the predicted values and average marginal effects of heart disease by COVID-19 diagnosis status are reported.

**Appendix Table 4. Sensitivity analysis using 1%-99% of dependent variable: Total medical costs associated with heart disease by COVID-19 diagnosis and race ethnicity, 2021.^a^**

|  | All | Without COVID-19 diagnosis | With COVID-19 diagnosis | With COVID-19 vs. Without COVID-19^c^ |
| --- | --- | --- | --- | --- |
| All races |  |  |  |  |
| Without heart disease | 10,659 | 9,958 | 19,338 | 9,380*** |
|  | (10,547 - 10,771) | (9,862 - 10,055) | (18,863 - 19,814) | (8,947 - 9,814) |
| With heart disease | 18,952 | 17,774 | 33,537 | 15,763*** |
|  | (18,626 - 19,278) | (17,448 - 18,100) | (32,114 - 34,959) | (14,323 - 17,202) |
| Difference^b^ | 8,293*** | 7,816*** | 14,198*** | 6,382*** |
|  | (7,972 - 8,614) | (7,491 - 8,141) | (12,770 - 15,626) | (4,923 - 7,842) |
| Observation | 998,085 | 923,480 | 74,605 | 998,085 |
| Non-Hispanic White |  |  |  |  |
| Without heart disease | 11,510 | 10,753 | 21,235 | 10,482*** |
|  | (11,380 - 11,640) | (10,639 - 10,866) | (20,570 - 21,900) | (9,847 - 11,117) |
| With heart disease | 19,137 | 18,015 | 33,553 | 15,538*** |
|  | (18,678 - 19,597) | (17,552 - 18,479) | (31,470 - 35,636) | (13,416 - 17,661) |
| Difference^b^ | 7,628*** | 7,262*** | 12,318*** | 5,056*** |
|  | (7,165 - 8,090) | (6,793 - 7,732) | (10,185 - 14,452) | (2,875 - 7,237) |
| Observation | 461,947 | 428,580 | 33,367 | 461,947 |
| Non-Hispanic Black |  |  |  |  |
| Without heart disease | 12,799 | 11,734 | 25,777 | 14,043*** |
|  | (12,580 - 13,018) | (11,545 - 11,923) | (24,561 - 26,993) | (12,865 - 15,221) |
| With heart disease | 24,730 | 23,029 | 45,453 | 22,425*** |
|  | (23,853 - 25,606) | (22,144 - 23,914) | (41,637 - 49,270) | (18,534 - 26,315) |
| Difference^b^ | 11,931*** | 11,295*** | 19,677*** | 8,382*** |
|  | (11,060 - 12,801) | (10,412 - 12,178) | (15,745 - 23,609) | (4,358 - 12,406) |
| Observation | 186,775 | 172,607 | 14,168 | 186,775 |
| Hispanic |  |  |  |  |
| Without heart disease | 4,867 | 4,560 | 8,194 | 3,634*** |
|  | (4,800 - 4,934) | (4,498 - 4,622) | (7,834 - 8,554) | (3,275 - 3,993) |
| With heart disease | 12,106 | 11,375 | 20,034 | 8,659*** |
|  | (11,436 - 12,776) | (10,683 - 12,067) | (17,471 - 22,597) | (6,005 - 11,313) |
| Difference^b^ | 7,239*** | 6,815*** | 11,840*** | 5,025*** |
|  | (6,566 - 7,912) | (6,120 - 7,510) | (9,256 - 14,423) | (2,350 - 7,699) |
| Observation | 152,207 | 139,353 | 12,854 | 152,207 |

^a^ The total medical costs were restricted between 1% and 99% of the values to test whether the results were driven by the outliers. A generalized linear model with a family of gamma and log links with was used. All models were adjusted to patients’ age, sex, race categories (for all only), COVID-19 infection status, and comorbidities. The average predicted total medical costs with 95% CI for individuals with and without heart disease are reported. The robust standard errors were used. The differences and 95% CI in the predicted total medical costs for individuals with heart disease and without heart disease were reported. Interaction terms of heart disease indicator, race categories, and COVID-19 diagnosis indicator were used to calculate the predicted values for the NH-White, NH-Black, and Hispanic by COVID-19 diagnosis status.

^b^ Difference in costs for individuals with heart disease vs. those without heart disease. *** *P<*0.001, ** *P<*0.01, * *P<*0.05

^c^ Differences with 95% CI in the predicted values and average marginal effects of heart disease by COVID-19 diagnosis status are reported.
